# Selected Papers on Operative and Clinical Surgery

**Published:** 1903-03

**Authors:** 


**Selected Papers on Operative and Clinical Surgery.** By Sir William Stokes, M.D., M.Ch. Edited by William Taylor, B.A., M.B. Pp. xxv., 484. London: Baillière, Tindall & Cox. 1902.

The greater part of this book consists of a number of papers, either published in journals or read before Societies by the late Sir William Stokes. The papers are well written, and amongst them are a number of very interesting clinical cases. As, however, many of them date back to the seventies, and some even to the sixties, it will be found that their interest lies in their expression of the surgical principles of a generation past, rather than in that of the surgery of to-day.

In the chapter on aneurysm no mention is made of excision of the sac, or ligation close above the sac in the spontaneous form of the disease, compression being the method of treatment mainly advocated. It seems strange that Sir William Stokes should have only seen one traumatic aneurism of the radial artery. We should have thought this one of the arteries most frequently affected.

Davy's lever is strongly recommended for arresting hemorrhage in amputation of the hip-joint, although from the account of the cases it does not seem to have been entirely satisfactory. The amputation by the anterior racket incision is somewhat summarily disposed of.

It seems strange that the case of rupture of the female bladder, recorded in chapter ix., was not operated on. The symptoms appeared sufficiently obvious, yet the patient was watched for five days getting steadily worse until her death, and nothing done.

In the chapter on operations of the thyroid gland, the question of the advisability of removal of the whole organ is discussed,—a question now long ago settled. In removal of the tongue, the ecraseur is advocated. We cannot help agreeing with Treves, that this method has "about it a distinct air of senility." Pins are advised in the operation for hare-lip. We imagine few surgeons use them now. Some of the plates in the book on this and other sections, if accurately reproduced, show most wonderfully good cosmetic results. Median lithotomy, as the operation of election, is hardly in accordance with present-day teaching.

It will be seen from these few remarks that the interest of much of the writing is historical. Towards the end of the book are a number of addresses on surgery, given by the author at various times. The last chapter records some of his South African experiences, and at the beginning is a short but interesting introductory memoir by Ogston.

